# Department of Practice of Medicine

**Published:** 1894-06

**Authors:** 

**Affiliations:** Professor of Pathology and Lecturer on Mental and Nervous Diseases in the Medical Department of the University of Texas, Galveston


### DEPARTMENT OF PRACTICE OF MEDICINE.

EDITED BY PROF. ALLEN J. SMITH, M. D., GALVESTON,
Professor of Pathology and Lecturer on Mental and Nervous Diseases in the Medical Department of the University of Texas, Galveston.

ELECTRICITY IN THE TREATMENT OF GOITRE.—Dr. J. L. Howard (*American Practitioner and News,* February 10, 1894) states that during the past eighteen months sixteen cases of goitre have been treated by electrophoresis of iodine, at the clinic of the University of Louisville. Of these, seven have been discharged as cured; five are under treatment, all improving; and five have been lost sight of. The writer states that beneficial effects are to be obtained in any of the hypertrophic varieties of goitre, acute or chronic, by this method. In applying the method, a cup-shaped electrode of gutta-percha is employed as the positive element; in this cup is placed a small pledget of absorbent cotton moistened with a solution of sodium chloride, and containing eight or ten drops of Churchill's tincture of iodine. This is placed over the goitre, with the kathode upon the back of the neck, and the current is turned on gradually until the patient is able to taste iodine. Each application should last perhaps ten to fifteen minutes, and should be repeated two or three times a week, or daily, as the case requires. The writer refers to the fact that the iodine is forced through the tissues by the electric current, and that the negative electrode having been wet with a starch solution, will become blue from the formation of the iodide of starch when the current is passed.

TREATMENT OF ERYSIPELAS WITH STRONG ALCOHOL.—Dr. A. J. Ochsner, of Chicago, Ill. (*Kansas City Medical Index*, April, 1894), following the practice of Behrand, of Germany, recommends that in cases of erysipelas the surface be vigorously scrubbed three or four times daily with absolute alcohol. It is claimed that it will cut short the attack in from three to five days, the temperature being decidedly reduced in twenty-four hours, the blush almost immediately fading, and the patient feeling practically well after forty-eight hours.

